# Multidimensional factors of depressive symptoms among Chinese undergraduate students: a cross-sectional study using binary logistic regression

**DOI:** 10.3389/fpsyt.2025.1546197

**Published:** 2025-08-13

**Authors:** Wenjia Chen, Jianfei Bai, Haitao Niu, Qingying Zhu, Xiuhan Zhao, Yanyu Dong

**Affiliations:** ^1^ School of Physical Education, China University of Mining and Technology, Xuzhou, China; ^2^ School of Physical Education, Shandong University, Jinan, China; ^3^ Yanjing Medical College, Capital Medical University, Beijing, China

**Keywords:** college students, depressive symptoms, lifestyle, behavioral patterns, psychosocial factors, logistic regression

## Abstract

**Introduction:**

This cross-sectional study investigates the interplay of lifestyle, behavioral, and psychosocial factors in predicting depressive symptoms among Chinese college students (N=508) using binary logistic regression.

**Methods:**

Participants were recruited from four geographically diverse provinces (Eastern: Shandong; Western: Shaanxi, Sichuan; Southern: Hainan) across 8 universities (5 comprehensive universities, 3 specialized institutions), with balanced urban (n=245, 48.22%) and rural (n=263, 51.78%) representation. Standardized scales assessed depressive symptoms (PHQ-9), perceived stress (PSS), academic procrastination (PASS), physical activity (PARS-3), cyberbullying victimization (CVQ), and alcohol use (AUDIT).

**Results:**

Results revealed significant negative associations of sleep duration and physical activity with depressive symptoms (p<0.05), while academic procrastination, cyberbullying, perceived stress, and alcohol consumption showed significant positive predictive effects (p<0.01).

**Discussion:**

Despite limitations of convenience sampling and cross-sectional design, findings suggest a complex interplay of multidimensional factors in college students’ mental health. Early screening and comprehensive interventions targeting lifestyle, behavioral, and psychosocial domains are recommended. Further longitudinal and experimental studies are warranted to elucidate causal pathways and inform targeted prevention strategies in university settings.

## 1 Introduction

Depression has emerged as a major global health challenge, with the World Health Organization projecting it to be the second leading cause of disease burden by 2030, surpassed only by ischemic heart disease (1). College students, navigating a critical developmental stage, face numerous stressors such as environmental changes, interpersonal relationships, academic pressures, and career prospects, rendering depression a significant risk factor for their physical and mental well-being (2). Numerous studies have consistently demonstrated substantially higher rates of depression among Chinese college students compared to the general population (3, 4). Depression among college students has garnered increasing attention as a pressing public health issue, necessitating effective prevention and intervention strategies. This study focuses specifically on depressive symptoms, which refer to specific manifestations such as low mood, loss of interest, sleep disturbances, and cognitive difficulties that can be measured on a continuum (5). These symptoms may occur in various mental health conditions and do not necessarily indicate a clinical depressive disorder. In contrast, depressive mood disorders are clinical diagnoses that require specific criteria including symptom duration, severity, and functional impairment (1). Depressive symptoms not only profoundly impact students’ academic performance, daily functioning, and social interactions but may also precipitate extreme behaviors, including suicide (5). Recent epidemiological studies have documented the substantial burden of depressive symptoms among Chinese university students. A systematic review of 84 studies during the COVID-19 pandemic found a pooled prevalence of 26.0%, with significantly higher rates among females (30.8%) and postgraduates (29.3%) (6). Similarly, Xu et al. (2022) reported that 24.8% of university freshmen experienced depressive symptoms, with possible avoidant personality disorder magnifying the association between bullying victimization and depression (7). However, these studies predominantly relied on convenience sampling and cross-sectional designs, limiting causal inference. Moreover, most investigations examined risk factors in isolation, failing to simultaneously assess multiple domains of influence (lifestyle, behavioral, and psychosocial factors) that may independently contribute to depression risk. The considerable heterogeneity in assessment tools and cutoff scores across studies further complicates the understanding of depression’s multifactorial nature in this population, highlighting the need for theory-driven, comprehensive approaches that examine multiple risk domains within a unified framework.

Traditionally, depression has been conceptualized through a biomedical lens, emphasizing clinical symptomatology and biological etiologies, with pharmacotherapy serving as the primary treatment modality (8). However, the advent and evolution of the biopsychosocial model have catalyzed a paradigm shift, recognizing the complex interplay of biological, psychological, and social factors in the onset and progression of depression (9). This multidimensional perspective underscores the importance of examining the role of individual lifestyles, behavioral patterns, and social contexts in shaping mental health outcomes. Indeed, accumulating evidence suggests that lifestyle factors such as physical activity and sleep duration (10, 11), behavioral tendencies like academic procrastination and alcohol consumption (12, 13), and psychosocial variables including perceived stress and cyberbullying victimization (14, 15) may significantly influence depressive symptomatology among college students. Nevertheless, a comprehensive understanding of the key determinants of depression in this population remains elusive, necessitating a systematic exploration of the multifaceted risk and protective factors and their underlying mechanisms (16).

Elucidating the complex etiological pathways of depression is of paramount importance, given its multifactorial nature and the paucity of research integrating diverse theoretical perspectives and methodologies (17). College students, undergoing rapid physiological and psychological development while simultaneously confronting myriad environmental transitions and adaptive challenges, represent a unique population requiring a nuanced, multidimensional approach to understanding the interplay of lifestyle, behavioral, and psychosocial factors in the context of depression (18). Such an endeavor holds significant theoretical and practical implications, enabling the development of targeted, evidence-based strategies for mental health education, counseling services, and crisis intervention tailored to the specific needs of college students (19). However, extant literature has largely focused on isolated factors within specific domains, with few studies attempting to systematically interrogate the complex interactions among lifestyle, behavioral, and psychosocial variables in relation to college students’ depression (20). To address this critical gap, the present study aims to recruit a nationally representative sample of Chinese college students and construct a comprehensive analytical framework encompassing lifestyle, behavioral, and psychosocial dimensions, grounded in the biopsychosocial model. By employing binary logistic regression to empirically investigate the interactive effects of these multifaceted factors on depressive symptomatology, this research seeks to provide valuable insights and recommendations for curbing the escalating prevalence of depression among college students and promoting their holistic well-being.

The biopsychosocial model, proposed by Engel, offers a compelling theoretical framework for systematically examining the multidimensional determinants of depression (9). This paradigm emphasizes the dynamic interplay of biological, psychological, and social factors in the etiology and progression of physical and mental health conditions, transcending reductionistic, single-cause explanations and situating individuals within a complex, ever-changing environmental context. Drawing upon this integrative perspective, the present study focuses on three key domains—lifestyle, behavioral patterns, and psychosocial factors—and their potential contributions to depressive symptomatology among college students.

Lifestyle factors, encompassing health-related behaviors such as diet, exercise, and sleep, play a crucial role in shaping mental well-being. Extensive research has demonstrated the protective effects of regular physical activity on emotional states and depressive symptoms (21). Conversely, sleep disturbances and disordered eating patterns have been positively associated with depressive symptomatology (22). Moreover, lifestyle factors are intimately linked to various physical health outcomes, such as cardiovascular diseases and metabolic disorders, which may indirectly influence depression through complex physiological and psychological pathways (23). Given that college students are at a critical juncture in establishing long-term lifestyle habits, investigating the relationship between these variables and depressive symptoms is of paramount importance.

Behavioral patterns, particularly maladaptive coping strategies, have been implicated in the development and maintenance of depressive symptomatology. Academic procrastination, a prevalent phenomenon among college students, has been shown to significantly impact mental health outcomes. Procrastinatory behaviors often lead to diminished academic performance, self-criticism, guilt, and subsequent depressive symptoms (24). Similarly, alcohol consumption has been linked to dysregulation of neurotransmitter systems, particularly serotonergic pathways, which are implicated in the pathophysiology of depression (25). Maladaptive behavioral patterns often co-occur with lifestyle irregularities, synergistically contributing to the onset and exacerbation of depressive symptoms. Therefore, this study seeks to elucidate the relationship between academic procrastination, alcohol use, and depressive symptomatology among college students.

Psychosocial factors, reflecting the complex interplay between individual psychological processes and social contexts, have garnered significant attention in depression research. The stress-vulnerability model posits that stressful life events and individuals’ subjective appraisals thereof are critical precipitants of depressive episodes (26). Empirical evidence suggests that perceived stress (27) and experiences of cyberbullying victimization (28) are significantly associated with depressive symptoms among college students. The university setting presents myriad challenges, including environmental adaptation, academic demands, and interpersonal conflicts, which may exacerbate stress perceptions and precipitate psychological distress. Moreover, the pervasive use of digital technologies has given rise to new forms of interpersonal aggression, such as cyberbullying, which can erode self-esteem, deplete coping resources, and engender feelings of helplessness and despair (29). Therefore, this study aims to investigate the role of perceived stress and cyberbullying victimization, as salient psychosocial variables, in predicting depressive symptoms among college students.

While the above studies have primarily focused on clinical depression or depressive disorders, the present study specifically examines depressive symptoms as a continuous variable. This approach allows for the identification of subclinical manifestations that may serve as early warning signs and targets for preventive interventions, particularly relevant in non-clinical populations such as college students. Based on the aforementioned theoretical foundations and empirical evidence, this study proposes the following hypotheses:

H1: Lifestyle variables (sleep duration and physical activity) will be significantly negatively associated with depressive symptoms.

H2: Behavioral variables (academic procrastination and alcohol consumption) will be significantly positively associated with depressive symptoms.

H3: Psychosocial variables (perceived stress and cyberbullying victimization) will be significantly positively associated with depressive symptoms.

## 2 Materials and methods

### 2.1 Study design and participants

This study employed a cross-sectional survey design with undergraduate students from universities in four geographically diverse provinces (Shandong, Sichuan, Hainan, and Shaanxi). Due to practical constraints such as limited resources, time restrictions, and challenges in accessing a comprehensive sampling frame, convenience and snowball sampling techniques were employed instead of the ideal random sampling approach. Although this methodological choice may limit generalizability, the geographic diversity of our sample partially addresses this concern. Data were collected through an online questionnaire administered between April and June 2022. Graduate students initiated the recruitment process by disseminating electronic questionnaire links to undergraduate students within their networks. To enhance response rate and minimize social desirability bias, we issued an open letter prior to the formal test detailing the research objectives, emphasizing data confidentiality and participant anonymity. Additionally, we used validated scales with demonstrated reliability in Chinese populations. The letter also emphasized that there were no right or wrong answers and encouraged respondents to provide truthful responses. A total of 560 questionnaires were collected, of which 508 were deemed valid after eliminating incomplete or inconsistent responses, resulting in an effective response rate of 90.71%. The sample consisted of 264 male and 244 female students, with a mean age of 20.67 years (SD = 1.99). Demographic characteristics of the participants were collected using a self-compiled personal information questionnaire, which included items such as gender, age, and student origin.

### 2.2 Measures

Dependent variable: The Chinese version of depressive Symptom Screening Scale (PHQ-9) was used to detect the depressive symptoms of college students. The scale consists of nine items that measure the nine DSM-IV criteria for the diagnosis of depressive episodes, using a 4-point scale (0=“ not at all “, 3=“ almost every day “) on a scale ranging from 0 to 27, with higher scores indicating more severe depressive symptoms. Referring to the standard cut-off value of 5 points, the two groups were divided into depressive symptoms group (≥5 points) and non-depressive symptoms group (<5 points). The scale has good reliability and validity, and the internal consistency coefficient Cronbach’s α in this study was 0.87 (30).

Lifestyle variables: Sleep time was measured by self-rated daily sleep hours; Participants’ physical activity levels were assessed using the 3-point Physical Activity Rating Scale (PARS-3), which divides individuals into low, medium, and high physical activity groups based on their self-reported frequency and intensity of exercise (31).

Behavioral variables: This study focuses on two typical problem behaviors: academic procrastination and drinking. The Procrastination Scale-Students (PASS) developed by Solomon and Rothblum (32) consists of 19 items on a 5-point scale (1=“ highly inconsistent “, 5=“ Very much “). Alcohol consumption was assessed using the Alcohol Use Disorders Identification Test (AUDIT), a 10-item screening tool developed by the World Health Organization (33). The AUDIT consists of three domains: items 1–3 assess alcohol consumption patterns; items 4–6 evaluate dependence symptoms; and items 7–10 measure alcohol-related problems. Items 1–8 are scored on a 5-point scale (0-4), while items 9–10 use a 3-point scale (0, 2, 4). The higher AUDIT score indicating more problematic alcohol use.

Psychosocial variables: This study selected perceived stress and cyberbullying as representative psychosocial factors. The Perceived Stress Scale (PSS) compiled by Cohen (34) consists of 14 items and is scored by 5 points (0=“ never “, 4=“ very frequently “). The higher the score, the higher the perceived stress level. The revised Cyber Victimization Questionnaire (35) was used for investigation, which included 15 items from 5 dimensions, including harassment, defamation, impersonation, disclosure of privacy, and social exclusion. A 5-point score was adopted (1=“ never “, 5=“ Always “), with a higher score indicating more severe cyberbullying.

### 2.3 Statistical methods

SPSS 22.0 was used for data processing and analysis. Firstly, descriptive statistics of demographic variables are carried out. Secondly, the correlation analysis of independent variables and dependent variables is carried out to investigate the correlation between each variable. Third, with “depressive symptoms or not” as the dependent variable (1= depressive symptoms, 0= non-depressive symptoms) and including demographic variables, lifestyle, behavioral habits and psychosocial factors as independent variables, binary Logistic regression was used to analyze the impact of each factor on depression of college students and test the research hypothesis. All statistical tests were two-sided with significance level α=0.05.

## 3 Results

### 3.1 Participant characteristics and prevalence of risk factors

Among 508 college students, the detection rate of depressive symptoms was 48.72%. Among the 508 participants, 11.2% (n=57) reported sleeping less than 6 hours daily, 73.4% (n=373) slept 6–8 hours, 15.4% (n=78) slept more than 8 hours. Regarding physical activity, 59.4% (n=302) were classified as having low physical activity levels, 10.4% (n=53) as moderate, and 30.2% (n=153) as high. For behavioral factors, the mean academic procrastination score was 29.17 (SD=8.71). The mean AUDIT score was 10.74 (SD=9.76), with 57.5% (n=292) exceeding the hazardous drinking threshold (≥8). For psychosocial factors, the mean perceived stress score was 27.19 (SD=7.06). The mean item score for cyberbullying victimization was 3.76 (SD=1.73). These descriptive findings reveal a high prevalence of low physical activity (59.4%) and hazardous drinking (57.5%) among participants. The differences in depressive symptoms among college students with different demographic variables are shown in **Table 1**. The results showed that there was no significant difference in the depressive symptoms detection rate among different genders, ages, and grades (p>0.05).

**Table 1:** Comparison of depressive symptoms detection rates among college students with different demographic characteristics.

Demographic variable	Category	Number of cases	Depressive symptoms detection rate (%)	χ²	df	p	Cramer’s V
Gender	Male	264	45.83	1.96	1	0.16	0.06
	Female	244	52.00				
Grade	Freshman	163	49.07	6.58	5	0.25	0.11
	Sophomore	95	53.68				
	Junior	89	57.30				
	Senior	161	42.86				
Place of Origin	Urban	245	52.00	1.73	1	0.19	0.06
	Rural	263	45.62				
Only a Child or Not	Yes	65	49.23	0.01	1	0.94	0.01
	No	443	56.00				

### 3.2 Correlation analysis: correlation between lifestyle, behavioral habits, psychosocial factors and depressive symptoms

The correlation coefficients and descriptive statistical results among variables are shown in **Table 2**. Correlation analysis indicated that daily sleep duration and physical activity were negatively correlated with depressive symptoms, while alcohol consumption, academic procrastination, and cyberbullying victimization were positively correlated with depressive symptoms. Additionally, perceived stress levels were also positively correlated with depressive symptoms.

**Table 2:** The relationship between lifestyle, behavioral habits, psychosocial factors and depressive symptoms.

Variables	1	2	3	4	5	6	7
1. Depressive Symptoms	1						
2. Daily Sleep Duration	-0.22**	1					
3. Alcohol Consumption	0.25**	-0.01	1				
4. Physical Activity	-0.13**	0.14**	0.26**	1			
5. Academic Procrastination	0.29**	-0.10**	0.09*	-0.01	1		
6. Cyberbullying	0.28**	-0.06	0.39**	-0.01	0.15**	1	
7. Perceived Stress	0.34**	-0.16**	0.06	-0.05	0.37**	0.07	1

*p < 0.05, **p < 0.01.

### 3.3 Regression analysis: the predictive effect of multiple factors on depressive symptoms in college students

Taking “depressive symptoms” as the dependent variable and including 6 independent variables significantly related to depressive symptoms, binary logistic regression was used to analyze the multi-dimensional influencing factors of depressive symptoms in college students. The results are shown in **Table 3**. The analysis results showed: (1) Among lifestyle variables, sleep duration (β=-0.67, OR=0.51) and physical activity (β=-0.01, OR=0.99) had significant negative predictive effects on depressive symptoms in college students (p<0.05), that is, the longer the sleep duration and the greater the amount of exercise, the lower the risk of depressive symptoms. H1 is partially verified. (2) Among behavioral variables, academic procrastination (β=0.03, OR=1.03) and alcohol consumption (β=0.04, OR=1.04) had significant positive predictive effects on depressive symptoms in college students (p<0.05 and p=0.001, respectively), that is, the higher the level of academic procrastination and alcohol consumption, the greater the risk of depressive symptoms. H2 is verified. (3) Among psychosocial variables, perceived stress (β=0.09, OR=1.10) and cyberbullying victimization (β=0.25, OR=1.29) both had significant positive predictive effects on depressive symptoms in college students (p<0.01), that is, the higher the level of perceived stress and experience of cyberbullying, the greater the risk of depressive symptoms. H3 is verified. In conclusion, lifestyle variables (sleep duration and physical activity), behavioral variables (academic procrastination and alcohol consumption), and psychosocial variables (perceived stress and cyberbullying victimization) can all significantly predict depressive symptoms in college students, with psychosocial variables having the most prominent predictive effect. Supplementary analyses revealed no significant statistical interactions between these factors, suggesting they contribute independently to depression risk.

**Table 3:** Logistic regression analysis of multiple factors on depressive symptoms of college students (n=508).

Variables	β	B	S.E.	Wald	df	Sig.	Exp(B)
Daily Sleep Duration	-0.34	-0.67	0.22	9.54	1	0.01	0.51
Physical Activity	-0.32	-0.01	0.01	8.62	1	0.01	0.99
Academic Procrastination	0.28	0.03	0.01	6.65	1	0.01	1.03
Cyberbullying	0.41	0.25	0.08	8.88	1	0.01	1.29
Perceived Stress	0.64	0.09	0.02	25.84	1	0.000	1.10
Alcohol Consumption	0.40	0.04	0.01	10.72	1	0.01	1.04
Constant	–	3.20	0.77	17.48	1	0.000	0.04

## 4 Discussion

### 4.1 Interpretation of key findings

The present study revealed a high prevalence of depressive symptoms among Chinese college students, with 48.9% of participants meeting the criteria for depressive symptoms based on the PHQ-9 cut-off score. This finding is notably higher than the 23.8% prevalence reported by Lei et al. (36) and the 22.9% prevalence among Chinese college students found by Xu et al. (4). These results underscore the severity of the mental health challenges faced by Chinese college students and align with the growing concern regarding youth depression in recent years (2). However, it is important to note that the specific prevalence rates reported in the literature vary considerably, which may be attributable to differences in depression assessment tools, diagnostic criteria, survey timing, sample characteristics, and other methodological factors (20, 37). Future research should prioritize the standardization of depression assessment criteria and the implementation of regular national epidemiological surveys to enable more accurate and dynamic monitoring of depression trends among college students.

Our findings both converge with and diverge from other East Asian studies. The high prevalence (48.72%) and lack of gender differences contrast with some regional studies but may reflect China’s unique educational pressures and evolving gender dynamics (38). The cyberbullying-depressive symptoms association appears consistent across East Asian contexts, suggesting a shared regional challenge. However, the weaker protective effect of physical activity compared to other Asian studies may reflect differences in sports infrastructure and cultural attitudes toward exercise (39). These variations underscore the need for culturally-adapted rather than imported intervention models.

Interestingly, the current study did not find significant associations between demographic characteristics, such as gender and grade level, and depressive symptoms among college students. This finding contrasts with some previous studies that have reported higher prevalence rates of depression among female college students compared to their male counterparts and a decreasing trend in depression prevalence with increasing grade level (40, 41). Several factors may contribute to these discrepancies. First, the convergence of gender roles in contemporary society, driven by the growing prominence of gender equality, may have narrowed the differences between male and female college students in terms of lifestyle, role expectations, coping styles, and other relevant factors, thereby attenuating gender differences in depression (42). Second, college students across all grade levels may face multiple, persistent stressors related to academics, interpersonal relationships, and future employment prospects, which could diminish grade-level differences in depression (43). However, it is worth noting that some studies have found an increasing trend in depression prevalence among Chinese female college students as they progress through their academic years, potentially reflecting the unique social and cultural pressures experienced by women (3). These findings underscore the importance of considering the specific social and cultural context when examining the relationship between demographic factors and depression, and highlight the need for further exploration using qualitative research methods.

Among the investigated predictors, the present study found significant negative associations between sleep duration, physical activity, and depressive symptoms among college students. These results align with previous research demonstrating the link between sleep disturbances, sedentary behavior, and other lifestyle factors and increased risk of depression (10, 11). The interaction theory of the ternary system posits that the development of depression is closely related to dysfunction of the hypothalamic-pituitary-adrenal axis, and sleep deprivation can disrupt the secretion of melatonin, leading to endocrine rhythm disturbances and subsequent depressive symptoms (44). Moreover, sleep deprivation has been shown to impair the amygdala-prefrontal circuit, weakening an individual’s cognitive control over emotions and exacerbating depressive symptoms (45). In contrast, regular physical exercise has been found to improve endocrine function, promote the secretion of mood-enhancing neurotransmitters such as beta-endorphins and serotonin, and alleviate depressive symptoms (46). Other studies have suggested that physical exercise may also help reduce stress and enhance self-esteem and self-efficacy, thereby serving as a protective factor against depression (47). These findings underscore the importance of addressing sleep management and exercise habits among college students as potential avenues for preventing and managing depression through lifestyle modifications.

The current study also confirmed the significant predictive effect of academic procrastination, a typical maladaptive coping strategy, on depressive symptoms among college students. This finding is consistent with previous research in this domain. Using structural equation modeling, Eisenbeck et al. demonstrated that academic procrastination exacerbates depressive symptoms in college students by increasing perceived stress and diminishing self-esteem (48). Similarly, Zhang et al. found that perfectionism personality traits mediate between academic procrastination and suicidal ideation, which is often associated with depression (49). The behavior-emotional response theory suggests that academic procrastination is often accompanied by negative emotions such as shame and guilt, which can subsequently be internalized, leading to the development of depressive symptoms (50). Furthermore, chronic procrastination can result in academic underachievement and feelings of frustration, which may also contribute to the onset of depression. These findings suggest that academic procrastination may adversely impact college students’ mental health through a vicious cycle involving cognition, emotion, and behavior. Interventions aimed at enhancing learning skills, promoting effective time management strategies, and fostering a healthy self-evaluation system may prove beneficial in reducing procrastination and its associated negative outcomes.

Among the psychosocial factors investigated, the present study reaffirmed the strong association between perceived stress and depressive symptoms. This finding aligns with Lazarus and Folkman’s seminal cognitive-interactive stress theory (51), which posits that when individuals encounter stressful events and perceive the environmental demands as exceeding their coping resources, they experience stress perception, which can subsequently lead to feelings of helplessness and depression. College students are navigating a period of significant change, often confronted with stressful life events across various domains, including academics, interpersonal relationships, and daily life. Given their limited coping abilities, college students are particularly susceptible to stress responses (52). Moreover, during this developmental stage, self-identity is still malleable and easily influenced by negative evaluations, further contributing to heightened stress perceptions (53). These findings underscore the importance of cultivating positive coping styles, enhancing problem-solving skills, and promoting mental resilience as key strategies for addressing depression among college students.

Furthermore, the current study confirmed cyberbullying victimization as a significant psychosocial risk factor for depressive symptoms among college students, echoing the conclusions of previous research (54, 55). Cyberbullying can erode an individual’s self-esteem and sense of belonging, deplete their coping resources, and engender feelings of helplessness and depression (29). Attribution theory suggests that the anonymity and public nature of cyberbullying can easily lead to self-attribution and self-blame among victims, further exacerbating depressive symptoms (56). Additionally, cyberbullying often disrupts real-life relationships and deprives individuals of social support, thereby increasing the risk of depression (57). For contemporary college students, online interpersonal communication has become an integral part of their social interactions, and the detrimental effects of cyberbullying cannot be overlooked. These findings emphasize the urgent need to strengthen online behavior education, foster a positive online environment, and establish effective anti-bullying mechanisms to mitigate the negative impact of cyberbullying on college students’ mental health.

Further analysis of standardized regression coefficients (β) and odds ratios reveals a clear hierarchy among the investigated predictors. Perceived stress emerged as the strongest predictor (β = 0.64), followed by cyberbullying victimization (β = 0.41) and alcohol consumption (β = 0.40), suggesting that psychosocial factors and problematic drinking constitute the primary drivers of depressive symptoms in this population. Notably, while cyberbullying showed a moderate standardized coefficient, it demonstrated the largest odds ratio (OR = 1.29), indicating substantial practical impact on depressive symptom risk. Among protective factors, daily sleep duration (β = -0.34, OR = 0.51) and physical activity (β = -0.32) showed comparable standardized effects, though sleep duration exhibited a more pronounced protective effect based on its odds ratio. In contrast, behavioral factors such as academic procrastination (β = 0.28) demonstrated weaker associations, suggesting it may play a secondary role in the depression framework. This hierarchical understanding has important implications for intervention prioritization: resources should primarily target stress management, alcohol use screening and cyberbullying prevention programs, while simultaneously promoting healthy lifestyle habits such as adequate sleep and regular physical activity. The relatively modest contribution of academic procrastination suggests that addressing this behavioral factor in isolation may be insufficient without concurrent attention to the more influential psychosocial and substance use determinants.

The observed associations likely involve bidirectional mechanisms. The sleep-depression relationship exemplifies this complexity: sleep deprivation increases depression risk through HPA axis dysregulation, while depression disrupts sleep architecture via altered circadian rhythms, creating a self-perpetuating cycle (39). Similarly, perceived stress may operate through inflammatory processes and prefrontal-limbic circuit alterations. Academic procrastination functions both as consequence (reduced motivation) and precipitant (increased stress) of depression (58). These bidirectional relationships suggest that early intervention at any point in these cycles may prevent progression to clinical depression.

In summary, the present study demonstrates that depressive symptoms among college students are associated with a complex interplay of lifestyle factors, behavioral patterns, and psychosocial variables. These findings expand upon previous research on the determinants of depression and provide empirical support for the role of biological rhythm disturbances, maladaptive coping strategies, and adverse social environments in the development of depressive symptoms among college students. Engel’s biopsychosocial model offers a compelling framework for understanding this multifaceted interaction (9). Individuals are in constant, dynamic interaction with their environment, and biological functions, psychological states, and social situations reciprocally influence each other, collectively shaping health and disease outcomes (59, 60). Consequently, effective prevention and management of depression among college students necessitates a concerted effort addressing biological, psychological, and social dimensions. This may involve cultivating positive psychological qualities, promoting healthy lifestyles, and optimizing environmental support systems to ultimately foster the holistic well-being of college students (61). Achieving this goal requires the collaborative efforts of individuals, educational institutions, families, and society at large to establish a comprehensive, multidimensional mental health promotion framework.

Our hierarchical findings suggest a tiered intervention approach. Primary prevention should prioritize universal stress management programs integrated into academic curricula. Secondary interventions should target cyberbullying through both technological solutions (reporting systems) and educational initiatives. Given limited resources, universities should focus on high-impact, scalable interventions: peer-led stress reduction programs, dormitory-based sleep hygiene initiatives, and digital mental health tools. The modest role of certain behavioral factors suggests resources may be better allocated to addressing psychosocial determinants.

### 4.2 Research implications

The present study offers several noteworthy theoretical innovations and practical implications. First, by incorporating multidimensional factors such as lifestyle, behavioral patterns, and psychosocial variables into a comprehensive analytical framework, this study enriches the understanding of the determinants of depression within the Chinese cultural context. The findings provide a valuable theoretical foundation for future research aimed at aligning with Chinese cultural norms and the developmental needs of Chinese youth. Second, this study pioneered a cross-disciplinary, integrative research paradigm that synthesizes multiple factors from diverse domains into a unified analytical framework. By employing quantitative empirical methods to examine the interactive effects of these factors on depressive symptoms among college students, this study lays the groundwork for future multilevel, multidimensional depression research. Third, the results of this study lend support to and expand upon the biopsychosocial etiological model of depression. The findings demonstrate that factors reflecting biological rhythms, psychological well-being, and social adaptation are closely associated with the risk of depressive symptoms among college students, highlighting the complex interactions between psychological, behavioral, and environmental factors. These results provide empirical evidence for the multicausal theory of depression and contribute to the refinement of the biopsychosocial etiological model. By focusing on the critical transitional period of college, this study identifies specific factors influencing depression among young adults and enhances the understanding of the mechanisms underlying the development of depressive symptoms in this population.

The findings of this study also have significant practical implications for the accurate screening, assessment, prevention, and intervention of depression among college students, ultimately promoting their comprehensive physical and mental well-being. First, the study identifies key risk factors, such as sleep disturbances, physical inactivity, academic procrastination, stress, and interpersonal challenges, which can serve as indicators for early screening and targeted interventions. Second, the study highlights potential intervention entry points, including the promotion of physical exercise, improvement of study habits, provision of psychological skills training, and enhancement of resilience. Third, the study emphasizes the need for a comprehensive prevention and management system that involves the collaborative efforts of educational institutions, healthcare providers, and youth organizations, as well as the support of families, communities, and media, to create a nurturing environment conducive to the mental health of college students. This multifaceted approach is crucial for curbing the rising prevalence of depression among college students. Finally, the study underscores the importance of fostering self-management skills and personal growth among college students by promoting healthy attitudes, developing effective coping strategies, and leveraging peer support to enhance overall well-being. By empowering students to take an active role in maintaining their mental health and cultivating a supportive campus culture, this study suggests that college students can achieve optimal physical and mental development.

### 4.3 Limitations and future directions

While the present study contributes valuable insights to the understanding of the determinants of depression among college students, several limitations should be acknowledged, and future research directions should be considered. First, the cross-sectional design of this study precludes the establishment of causal relationships or determination of temporal sequences between variables. For instance, while we found that poor sleep is associated with depressive symptoms, we cannot determine whether sleep disturbances lead to depression or vice versa, or whether both are influenced by unmeasured third variables. Future longitudinal studies are essential to elucidate the directionality and causal pathways of these relationships. More specifically, a fundamental limitation is that the relationships identified may be bidirectional - poor lifestyle factors may not only contribute to depressive symptoms but also be manifestations of existing depression. Our cross-sectional design measured current states rather than behavioral changes over time, yet detecting changes (e.g., deteriorating sleep or increasing alcohol use over 1–2 weeks) may be more valuable for early identification. Future studies should employ longitudinal designs or ecological momentary assessment to capture these temporal dynamics. Second, the use of convenience and snowball sampling methods may have introduced selection bias, potentially affecting the representativeness of our sample. Students with severe depressive symptoms might be underrepresented due to reduced participation motivation, while those with larger social networks might be over represented. Additionally, despite recruiting from four provinces, the lack of random sampling limits the generalizability to the broader Chinese college student population. Future studies should employ stratified random sampling to enhance external validity. Third, Our reliance on self-reported measures may introduce recall bias and social desirability bias, particularly for sensitive topics such as alcohol consumption and depressive symptoms. Future studies should incorporate objective measures, such as actigraphy for sleep and physical activity assessment, clinical interviews for depression diagnosis, and biomarkers for stress assessment. Moreover, Our study did not assess several potentially important factors including family environment (e.g., parental mental health, family conflict, socioeconomic status), genetic predispositions, childhood trauma, and personality traits. These unmeasured variables may confound the observed associations and should be included in future comprehensive models of depression risk. Future research should adopt a more comprehensive approach by incorporating genetic markers, family history assessments, and detailed environmental measures to develop more complete etiological models.

Despite these limitations, the present study serves as a valuable starting point for interdisciplinary and multilevel depression research among college students. Future investigations should prioritize the refinement of assessment tools, the innovation of research paradigms and methodologies, and the deepening of the systematic understanding of the mechanisms underlying the development of depressive symptoms among college students. Specifically, future studies should examine early warning signs of depression among college students, such as changes in sleep patterns, social withdrawal, and declining academic performance. Developing simple screening tools for early detection and researching how to build supportive campus environments through peer support and stress reduction programs will be crucial for preventing the progression to clinical depression. By doing so, robust scientific evidence can be generated to inform and support the implementation of effective strategies for maintaining the physical and mental well-being of college students.

## 5 Conclusions

Based on the biopsychosocial medical model and binary logistic regression method, this study investigated the associations between multiple factors including demographic, lifestyle, behavioral, and psychosocial variables and depression among college students. The results show that: (1) In terms of lifestyle, sleep duration and physical activity are significantly negatively associated with depression in college students, which aligns with literature on biological rhythm regulation and physical health in mental well-being; (2) Among behavioral factors, academic procrastination showed a significant positive association with depression in college students, suggesting that maladaptive coping strategies may be linked to increased depression risk; (3) Among psychosocial factors, perceived stress and cyberbullying were significantly positively associated with depression in college students, highlighting the importance of psychological stress and social adversity in depression risk. Supplementary analyses revealed no significant statistical interactions between these factors, suggesting they contribute independently to depression risk. In summary, this study found that multi-dimensional factors across lifestyle, behavioral, and psychosocial domains are significantly associated with depression among college students. While these factors operate independently rather than synergistically, their combined presence may have cumulative effects on mental health, providing insights for comprehensive prevention strategies targeting multiple risk factors simultaneously.

## Data Availability

The raw data supporting the conclusions of this article will be made available by the authors, without undue reservation.
